# Ameliorative Effects of Rhoifolin in Scopolamine-Induced Amnesic Zebrafish (*Danio rerio*) Model

**DOI:** 10.3390/antiox9070580

**Published:** 2020-07-03

**Authors:** Ion Brinza, Ahmed M. Abd-Alkhalek, Mohamed A. El-Raey, Razvan Stefan Boiangiu, Omayma A. Eldahshan, Lucian Hritcu

**Affiliations:** 1 Department of Biology, Faculty of Biology, Alexandru Ioan Cuza University of Iasi, 700506 Iasi, Romania; ion.brinza@student.uaic.ro (I.B.); razvan.boiangiu@student.uaic.ro (R.S.B.); 2 Faculty of Medicine, Al-Azhar University, Cairo 11651, Egypt; Ahmedahmed1.stu.1@azhar.edu.eg; 3 Department of Phytochemistry and Plant Systematics, Pharmaceutical Division, National Research Centre, Dokki, Cairo 12622, Egypt; ma.aziz@nrc.sci.eg; 4 Department of Pharmacognosy, Faculty of Pharmacy, Ain Shams University, Abbassia, Cairo 11566, Egypt; 5 Center of Drug Discovery Research and Development, Ain Shams University, Cairo 11566, Egypt

**Keywords:** rhoifolin, zebrafish, anxiety, memory, cholinergic function, oxidative stress

## Abstract

Rhoifolin (Rho) exerts many biological activities such as anticancer, antidiabetic, hepatoprotective, antirheumatic, antibacterial, and antiviral properties. The neuroprotective action of this compound has not been studied. The goal of this study was to investigate the improvement impact of Rho on scopolamine (Sco)-induced zebrafish anxiety, amnesia, and brain oxidative stress and to elucidate the underlying mechanisms involved. Zebrafish were treated with Rho (1, 3, and 5 μg/L) for nine consecutive days and were subsequently subjected to Sco (100 μM) 30 min before behavioral tests (novel tank diving test, Y-maze, and novel object recognition tests). Rho was isolated from *Chorisia crispiflora* (Malvaceae) leaves and identified by different spectroscopic techniques. To further assess the possible mechanisms of Rho in enhancing the memory capacities in zebrafish, the in vivo antioxidant status and acetylcholinesterase (AChE) activity was also evaluated. Rho from *Chorisia crispiflora* leaves was identified. Rho could alleviate anxiety, memory deficits, and brain oxidative stress in Sco-treated zebrafish and could regulate the cholinergic function by inhibiting the AChE activity. Our results demonstrated that Rho could be a promising candidate compound against anxiety and amnesia by restoring the cholinergic activity and the amelioration of brain oxidative stress.

## 1. Introduction

Alzheimer’s disease (AD) is the most common type of dementia and is a progressive neurodegenerative disorder resulting in memory impairment and cognitive dysfunction [1]. One of the essential elements in the development of dementia tends to be the immoderate reduction of acetylcholine (ACh) hydrolyzed by acetylcholinesterase (AChE) in the brain of AD patients [2]. A decrease of the ACh often follows cholinergic cell depletion in the basal forebrain. One approach is to inactivate AChE activity, a critical enzyme that cleaves synaptic ACh and stops neuronal signals [3]. Cholinesterase inhibitors decrease the ACh extrasynaptic metabolism, increase the neurotransmitter’s synaptic residence time, and improve postsynaptic stimulation. Preserved postsynaptic cholinergic mechanisms transform the modified signal into cognitive and behavioral effects [2]. Scopolamine (Sco) is a commonly used model for the study of demented-related diseases because it can induce memory and cognitive deficits. This compound was used for antagonizing muscarinic acetylcholine receptors involved in working memory [4]. Sco has been shown to induce learning impairment in zebrafish, supporting the use of zebrafish as a model for the study of cholinergic mechanisms underlying learning and memory, the evaluation and screening of potential water-soluble chemicals that could modulate learning and memory processes, and the assessment of genetic manipulation behavioral phenotypes with up- or down-regulated cholinergic systems [5].

Rhoifolin (Rho, apigenin 7-O-β-neohesperidoside) is a flavone glycoside belonging to the apigenin family [6]. It was isolated from different plant sources, such as *Rhus* plants [7], artichoke, tomatoes, bananas, and grapes. Additionally, it was detected in many parts and juices from various *Citrus* spp. in high amounts [8].

Several studies have shown that this flavone glycoside possesses a variety of biological activities, such as antidiabetic activity in differentiated 3T3-L1 adipocytes, as it showed a dose-dependent insulin-mimetic effect (0.001–5 μM) and enhanced tyrosine phosphorylation of insulin receptor-β, adiponectin secretion, and GLUT4 translocation [9]. Furthermore, Rho exhibited potent in vitro cytotoxicity with high selectivity against human epidermoid larynx and human cervical carcinoma cell lines (IC_50_: 5.9 and 6.2 μg/mL). It induced a promising effect against hepatocellular and fetal human lung fibroblast cancer cell lines with IC_50_: 22.6, 34.8, and 44.6 μg/mL, respectively [10]. Additionally, it exhibited potent anti-inflammatory activity at low doses in carrageenan-induced rat paw edema and abolished the prostaglandin E2 level. Increasing doses of Rho significantly reduced the tumor necrosis factor-α (TNF-α) release. It also elevated the total antioxidant capacity in a reverse dose order, with the highest ability obtained with the lowest dose tested [11]. The in vitro study showed that Rho could actively suppress the receptor activators of nuclear factor-kappa B (NF-kB) ligand-stimulated osteoclastogenesis, F-actin formation, hydroxyapatite resorption, and the gene expression of osteoclast-related genes [12]. Kuo et al. [13] reported that Rho could protect neurons against beta-amyloid peptide (Aβ)-mediated neurotoxicity. It showed 80.3% hepatoprotection at 20 mg/kg against carbon tetrachloride (CCl4)-induced toxicity in mice. The serum levels of alanine transaminase (ALT) and aspartate aminotransferase (AST) and the general state of the liver was kept close to normal [14]. It also exerted a protective effect on gamma irradiation-induced cardiac dysfunctions in albino mice by decreasing the toxic effect of radiation via diminishing the lipid peroxides level, improving the alterations in nitric oxide, lactate dehydrogenase, creatine kinase in plasma and tissue, and the plasma lipid profile [15]. It exhibited antihypertensive effects in conscious spontaneously hypertensive rats [6]. Besides, it exerted a specific inhibitory activity against *Escherichia coli* and was also found to cause a 13% inhibition of coxsackievirus B3 infection with an IC_50_ of 569.05 μM [16]. However, no studies have identified the memory-enhancing effect of Rho on cognitive impairments due to cholinergic blockade. Therefore, this study aimed to investigate whether Rho attenuates the Sco-induced cognitive deficits in zebrafish using different behavioral paradigms (novel tank diving test, Y-maze, and novel object recognition tests).

## 2. Materials and Methods

### 2.1. Plant Material

Leaves of *Chorisia crispiflora* (Malvaceae) were collected from the Zoo Garden, Giza, Egypt, in 2018. The material was authenticated by Prof. dr. Abdel Salam El Noyehy, Professor of Taxonomy, Faculty of Science, Ain Shams University, Cairo, Egypt, and a voucher specimen was deposited at the Pharmacognosy Department Herbarium (No. PHG-P-CC-317), Faculty of Pharmacy, Ain Shams University, Cairo, Egypt.

### 2.2. Rhoifolin Extraction and Isolation from Chorisia crispiflora

The air-dried leaves (1 kg) of *Chorisia crispiflora* were extracted at room temperature with 70% ethanol. The extract was concentrated using a rotary evaporator under reduced pressure at 52 °C until complete dryness. The residue was dissolved in a small amount of water, and the aqueous extract was then successively partitioned with n-hexane, ethyl acetate, and butanol. The left aqueous residue was then totally dried and re-extracted with methanol at 40 °C. Upon concentration of the methanol extract, a yellow amorphous powder of Rho (7.8 g) was precipitated.

### 2.3. Structure Elucidation of Rhoifolin (Apigenin 7-O-β neohesperidoside)

UV λmax (MeOH): 266, 336 nm.

1H-NMR, DMSO-d6 δ ppm: 7.91(2H, d, J = 8.8 Hz, H-2‵,6‵), 6.92 (2H, d, J = 8.8 Hz, H-3‵,5‵), 6.84 (1H, d, J = 2.0 Hz, H-8), 6.80 (1H, s, H-3), 6.33 (1H, d, J = 2.0 Hz, H-6), 5.08 (1H, singlet like, H-1‷), 5.20 (1H, d, J = 7.3 Hz, H-1‶), 1.16 (3H, d, J = 6.3 Hz,CH3-6‷).

**Figure 1:**
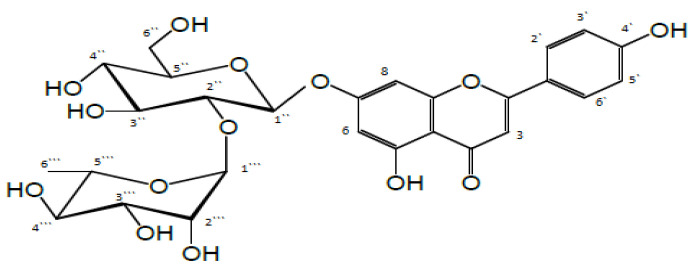
Chemical structure of rhoifolin (apigenin 7-O-β neohesperidoside).

13C-NMR, DMSO-d6 δ ppm: 182.1-C4, 164.4-C2, 162.6-C7, 161.7-C4‵, 161.1-C5, 157.1-C9,128.7-C2‵,6‵, 120.9-C1‵, 116.2C-3‵,5‵, 105.5-C10, 103.2-C3, 99.4-C6, 94.6-C8, Sugar proton: 100.5-C1 “, 98.2-C1‷, 77.6-C2 “, 77.4-C3 “, 76.8-C5 “, 72.3-C4‷, 71.0-C2‷, 70.8-C3‷, 71.1-C4 “, 68.8-C5‷, 60.9-C-6 “, 18.5-C-CH3, as shown in Figure 1.

### 2.4. Animals and Drug Administration

60 adult zebrafish (*Danio rerio*) of wild-type short-fin strain from both sexes (50:50 ratio), n = 10 per group, were purchased from an authorized commercial dealer (Pet Product S.R.L., Bucharest, Romania). The animals were held under adequate conditions of acclimatization at least one week before the experiments. Fish were kept in the light–dark cycle (14/10 h) photoperiod (lights on at 8:00 am), fed twice a day with Norwin Norvitall flake (Norwin, Gadstrup, Denmark), and housed in 24 L housing tanks (30 × 30 × 30 cm) at 26 ± 1 °C, pH = 7.5, dissolved oxygen at 7.20 mg/L, ammonium concentration < 0.004 ppm, and a conductivity of 500 μS. All tanks were maintained under constant mechanical filtration to avoid the accumulation of organic toxins. All animals were divided into the following groups: the control group, the scopolamine group (Sco, 100 μM, Sigma–Aldrich, Darmstadt, Germany), and three rhoifolin treatment groups (Rho: 1, 3, and 5 μg/L), the imipramine group (IMP, 20 mg/L, Sigma–Aldrich, Darmstadt, Germany, as a positive control within an novel tank diving test (NTT)) and the galantamine group (GAL, 1 mg/L, Sigma–Aldrich, Darmstadt, Germany, as a positive control within Y-maze and novel object recognition (NOR) tests). The doses of Sco, Rho, IMP, and GAL were chosen following a previous report [17]. Rho (1, 3, and 5 μg/L) was individually delivered to fish through transferring into a 500 mL glass for 1 h, once daily, whereas the Sco (100 μM) treatment was administered once independently by moving into a 500 mL glass 30 min before the behavioral tests [18]. This study was previously approved by the local board of ethics for animal experimentation (No. 15309/2019) and fully complied with the Directive 2010/63/EU of the European Parliament and of the Council of 22 September 2010 on the protection of animals. Efforts were made to reduce animal suffering and the number of animals utilized.

### 2.5. Behavioral Analysis

In our studies, a Logitech HD Webcam C922 Pro Stream camera (Logitech, Lausanne, Switzerland) recorded zebrafish swimming behavior, and the videos were analyzed using ANY-maze^®^ software (Stoelting CO, Wood Dale, IL, USA). Representative tracking images of the zebrafish locomotor activity from each group were obtained at the end of the analysis with ANY-Maze^®^ software.

#### 2.5.1. Novel Tank Diving Test (NTT)

The NTT is a specific test used for assessing anxiety in zebrafish, as described by Cachat et al. [19]. The apparatus used in the present study consists of a trapezoidal tank (1.5 L) (15.2 × 27.9 × 7.1 cm), equally divided into two horizontal sections (top and bottom). After 1 h of Rho treatment, each animal was moved individually to the testing apparatus, and the swimming behavior was recorded for 6 min. The time spent in top (s), time spent in top/bottom ratio, total distance traveled (m), and distance top/bottom ratio were the behavioral parameters evaluated in this test.

#### 2.5.2. Y-Maze Test

The response to novelty in zebrafish was assessed using the Y-maze test [20]. The position in the Y-maze test was considered an index of memory [21]. The apparatus consisted of a Y-maze glass aquarium (3 L) with three arms (25 × 8 × 15 cm). On the exterior walls of the Y-maze, recognizable geometric shapes such as squares, circles, and triangles were placed. The Y-maze arms were arbitrarily assigned: (i) the start arm, where fish started to investigate (always open), (ii) the novel arm, which was blocked during the first trial, but opened during the second trial, and the other arm (constantly open). The Y-maze center (neutral zone) was not counted. The task consisted of two trials to test the response to novelty and spatial memory, separated by 1 h between them. During the first trial (training, 5 min), 1 h after Rho treatment, just two arms of the Y-maze (the start and the other arm) could be explored, while the third arm (the novel arm) was obstructed. For the second trial, each fish was individually introduced in the start arm and had free access to all three arms for 5 min to assess the response to novelty. The time spent in each arm (% of total arm), total distance traveled (m), and turn angle (°) were the behavioral parameters evaluated in this test.

#### 2.5.3. Novel Object Recognition Test (NOR)

The NOR is a commonly used behavioral assay for the investigation of memory performance in zebrafish [22]. The experimental apparatus consists of a 20 L glass tank (30 × 30 × 30 cm) filled with 6 cm of water. Before training, each animal was habituated to the apparatus in the absence of the objects for 5 min twice a day (5 h interval between habituation sessions) over three consecutive days. On the fourth day, in the training phase, the animals were exposed to two identical red cubes for 10 min. After the training phase, the animals were submitted to a retention interval of 1 h. In the test phase, a new object (N, green cube) replaced one of the copies of the familiar objects (F, red cube), and the exploration time of each object was evaluated for 10 min. The exploratory time (s) and the preference percentages were the behavioral parameters evaluated in this test. The preference percentages were calculated as [time of exploration of N/time of exploration of F + time of exploration of N × 100].

### 2.6. Biochemical Parameters Assay

All zebrafish were euthanized (10 min immersion in ice water, 2–4 °C) until loss of opercular motions [23], and their whole brains were isolated for a biochemical parameters assay. The brains were gently homogenized in ice 0.1 M potassium phosphate buffer (pH 7.4), 1.15% KCl with Potter Homogenizer (Cole-Parmer, Vermon Hills, IL, USA). The resulting homogenate was centrifuged at 960× *g* for 15 min. The supernatant was used for the estimation of acetylcholinesterase (AChE), superoxide dismutase (SOD), catalase (CAT), glutathione peroxidase (GPX) specific activities, and the protein carbonyl and malondialdehyde (MDA) level.

#### 2.6.1. Determination of the AChE Activity

For the evaluation of acetylcholinesterase (AChE) activity, an earlier described method by Ellman et al. [24] was used. The final volume of the reaction mixture (600 μL) contained 0.26 M phosphate buffer with pH 7.4, 1 mM 5.5’-dithio-bis-2 nitrobenzoic acid (DTNB), and 5 mM acetylthiocholine chloride (ATC). The assay was started by adding supernatant and then following the development of the yellow color at room temperature at 412 nm for 10 min. Suitable controls for ATC’s non-enzymatic hydrolysis were performed. The enzyme activity was formulated as nmol of ACT/min per/mg of protein.

#### 2.6.2. Determination of the SOD Activity

For the determination of the activity of superoxide dismutase (SOD, EC 1.15.1.1), the method described previously by Winterbourn et al. [25] was applied. There were 100 mM TRIS/HCl (pH 7.8), 75 mM NBT, 2 μM riboflavin, 6 mM EDTA, and 200 μL supernatant in each 1.5 mL reaction mixture. The monitoring of the absorbance increases at 560 nm following the blue formazan output. One unit of SOD is classified as the amount needed to inhibit the NBT reduction rate by 50%. The enzyme activity was reported in units/mg protein.

#### 2.6.3. Determination of the CAT Activity

For the evaluation of the catalase (CAT, EC 1.11.1.6) activity, a formerly used method described by Sinha [26] was applied. 150 μL phosphate buffer (0.01 M, pH 7.0) and 100 μL supernatant were in the reaction mixture. The reaction was initiated by adding 250 μL H_2_O_2_ 0.16 M, incubated at 37 °C for 1 min, and then the reaction was stopped by adding 1 mL of dichromate: acetic acid reagent. The tubes were immediately kept in a boiling water bath for 15 min, and the green color formed during the reaction was read at 570 nm by using a spectrophotometer. Control tubes, devoid of the enzyme, were also processed in parallel. The activity of the enzyme is expressed as μmol of H_2_O_2_ consumed/min/mg protein.

#### 2.6.4. Determination of the GPX Activity

For the assessment of the glutathione peroxidase (GPX, E.C. 1.11.1.9) activity, a previous approach described by Sharma and Gupta [27] was used. A reaction mixture consisting of 1 mL 0.4 mM phosphate buffer (pH 7.0) containing 0.4 mM EDTA, 1 mL of 5 mM NaN_3_, 1 mL of 4 mM glutathione (GSH), and 200 μL of supernatant was pre-incubated at 37 °C for 5 min. Then, 1 mL of 4 mM H_2_O_2_ was inserted and incubated for another 5 min at 37 °C. The GSH excess was quantified using the 5,5’-dithiobis-2-nitrobenzoic acid (DTNB) method. One unit of GPX is specified as the amount of enzyme needed to oxidize for 1 nmol GSH/min. The enzyme activity was expressed as units/mg protein.

#### 2.6.5. Determination of the Protein Carbonyl Level

The extent of protein oxidation in the brain was assessed by measuring the content of protein carbonyl groups, using a method described by Oliver et al. [28] and modified through Luo and Wehr [29]. The supernatant fraction was split into two equal aliquots, each containing around 2 mg of protein. Both aliquots were precipitated using 10% trichloroacetic acid (TCA, *w*/*v*, final concentration). Another sample was treated with 2 N HCl, and another sample was treated with 0.2% (*w*/*v*) DNPH in 2 N HCl at an equivalent volume. Both samples were incubated at 25 °C and then stirred at intervals of 5 min. The results were expressed as nmol/mg protein.

#### 2.6.6. Determination of the MDA Level

The content of malondialdehyde (MDA), which is an indicator of lipid peroxidation, was measured via the usage of the approach previously described by Ohkawa et al. [30]. 200 μL of supernatant was applied and briefly mixed in 0.1 M HCl with 1 mL of 50% trichloroacetic acid in 0.1 M HCl and 1 mL of 26 mM thiobarbituric acid. Samples were held at 95 °C for 20 min after vortex mixing. Samples were then centrifuged for 10 min at 960× *g*, and the supernatants were read at 532 nm. The findings were presented as nmol/mg protein, as stated.

#### 2.6.7. Estimation of Protein Concentration

The protein content was estimated through Bradford’s dye-binding assay [31].

### 2.7. Data Analysis

Data are expressed as the mean ± standard error of the mean (SEM). The results were statistically analyzed by a one-way analysis of variance (ANOVA) followed by Tukey’s post hoc multiple comparison test, considering treatment as a factor. All analyses were performed by GraphPad Prism 8.0 software (GraphPad Software, Inc., San Diego, CA, USA), and the significance was set at *p* < 0.05.

## 3. Results and Discussion

### 3.1. Effects on Anxiety-Like Behavior in NTT and on Spatial Memory in Y-Maze and NOR Tests

**Figure 2:**
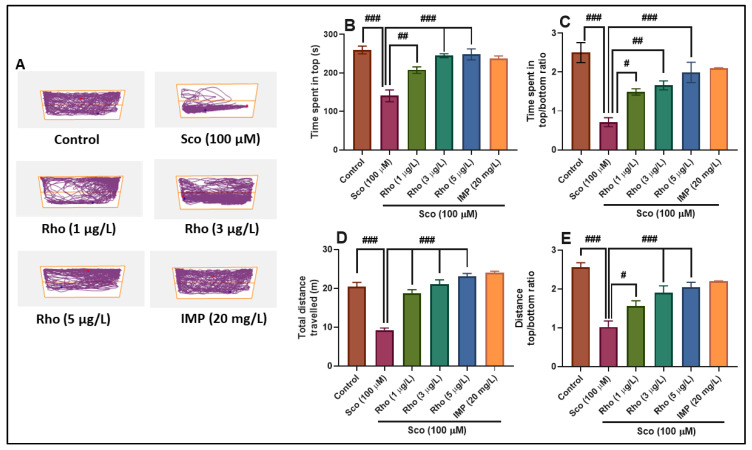
Rhoifolin (Rho: 1, 3, and 5 μg/L) improved the locomotion pattern and reduced anxiety in the novel tank diving test (NTT). (**A**) Representative locomotion tracking pattern of the control, scopolamine (Sco: 100 μM), rhoifolin (Rho: 1, 3, and 5 µg/L), and imipramine (IMP: 20 mg/L) treated groups. (**B**) Representation of the time spent in the top zone by zebrafish in the tank in different groups. (**C**) Representation of the time spent in top/bottom ratio in different groups. (**D**) Representation of the total distance traveled by zebrafish in the tank in different groups. (**E**) Representation of the distance top/bottom ratio in different groups. The values are means ± S.E.M. (n = 10). For Tukey’s post hoc analyses: (**B**) Control vs. Sco (100 μM): ### *p* < 0.0001, Sco (100 μM) vs. Rho (1 μg/L): ## *p* < 0.001, Sco (100 μM) vs. Rho (3 μg/L): ### *p* < 0.0001 and Sco (100 μM) vs. Rho (5 μg/L): ### *p* < 0.0001; (**C**) Control vs. Sco (100 μM): ### *p* < 0.0001, Sco (100 μM) vs. Rho (1 μg/L): # *p* < 0.01, Sco (100 μM) vs. Rho (3 μg/L): ## *p* < 0.001, and Sco (100 μM) vs. Rho (5 μg/L): ### *p* < 0.0001; (**D**) Control vs. Sco (100 μM): ### *p* < 0.0001, Sco (100 μM) vs. Rho (1 μg/L): ### *p* < 0.0001, Sco (100 μM) vs. Rho (3 μg/L): ### *p* < 0.0001, and Sco (100 μM) vs. Rho (5 μg/L): ### *p* < 0.0001; (**E**) Control vs. Sco (100 μM): ### *p* < 0.0001, Sco (100 μM) vs. Rho (1 μg/L): # *p* < 0.01, Sco (100 μM) vs. Rho (3 μg/L): ### *p* < 0.0001, and Sco (100 μM) vs. Rho (5 μg/L): ### *p* < 0.0001.

The representative locomotion tracking patterns (Figure 2A) illustrate the differences between the top and bottom zones in swimming traces within the NTT. Sco-treated groups exhibited a preference for the bottom zone, suggesting high levels of anxiety. In the NTT, the one-way ANOVA revealed a significant effect of the treatment on the time spent in top of the tank (F(5,54) = 17.24, *p* < 0.0001) (Figure 2B), on the time spent in top/bottom ratio (F(5,54) = 13.68, *p* < 0.0001) (Figure 2C), on the total distance traveled (F(5,54) = 38.21, *p* < 0.0001) (Figure 2D), and on the distance top/bottom ratio (F(5,54) = 16.54, *p* < 0.0001) (Figure 2E). Additionally, the Sco treatment decreased the time spent in the top zone of the tank (*p* < 0.0001) (Figure 2B) and time spent in top/bottom ratio (*p* < 0.0001) (Figure 2C) as compared to the control group. By decreasing the total distance traveled in the tank (*p* < 0.0001) (Figure 2D) and the distance top/bottom ratio (*p* < 0.0001) (Figure 2E), the Sco-administration induced a hypolocomotor effect as compared to the control group. In contrast, the Rho treatment avoided Sco induced-anxiogenic effects, especially at doses of 3 μg/L and 5 μg/L. IMP, used as a positive reference drug, evoked anxiolytic effects, as noticed by the behavioral parameters.

**Figure 3:**
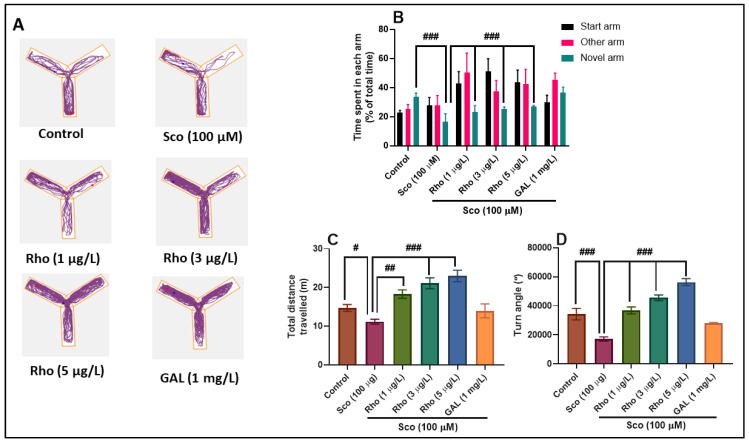
Rhoifolin (Rho: 1, 3, and 5 μg/L) improved the locomotion pattern and memory in the Y-maze test. (**A**) Representative locomotion tracking pattern of the control, scopolamine (Sco: 100 µM), rhoifolin (Rho: 1, 3, and 5 µg/L) and galantamine (GAL: 1 mg/L) treated groups. (**B**) Representation of the time spent in each arm (start, other, and novel arm) in different groups. (**C**) Representation of the total distance traveled by zebrafish in the tank in different groups. (**D**) Representation of the turn angle of zebrafish in the tank in different groups. Values are means ± S.E.M. (n = 10). For Tukey’s post hoc analyses: (**B**) Control vs. Sco (100 µM): ### *p* < 0.0001, Sco vs. Rho (1 µg/L): ### *p* < 0.0001, Sco vs. Rho (3 µg/L): ### *p* < 0.0001, and Sco vs. Rho (5 µg/L): ### *p* < 0.0001; (**C**) Control vs. Sco (100 µM): # *p* < 0.01, Sco vs. Rho (1 µg/L): ## *p* < 0.001, Sco vs. Rho (3 µg/L): ### *p* < 0.0001, and Sco vs. Rho (5 µg/L): ### *p* < 0.0001; (**D**) Control vs. Sco (100 µM): ### *p* < 0.0001, Sco vs. Rho (1 µg/L): ### *p* < 0.0001, Sco vs. Rho (3 µg/L): ## *p* < 0.0001, and Sco vs. Rho (5 µg/L): ## *p* < 0.0001.

The typical locomotion tracking pattern (Figure 3A) illustrates the differences in swimming traces among the Y-maze arms. It shows that the Sco-treated group traveled a greater distance in the other arm and less in the novel arm, indicating memory deficits. In the Y-maze test, the one-way ANOVA revealed a significant effect of the treatment on the time spent in each arm (F(5,54) = 16.88, *p* < 0.0001) (Figure 3B), on the total distance traveled (F(5,54) = 12.51, *p* < 0.0001) (Figure 3C), and on the turn angle (F(5,54) = 32.88, *p* < 0.0001) (Figure 3D). Additionally, the Sco administration resulted in decreasing the time spent in the novel arm (*p* < 0.0001) (Figure 3B), suggesting memory impairment. The administration of Sco affected locomotion as evidenced by the significant decrease of the total distance traveled (*p* < 0.01) (Figure 3C) and the turn angle (*p* < 0.0001) (Figure 3D), as compared to the control group. The Rho treatment greatly prevented the hypolocomotion and memory deficits caused by the Sco administration, as evidenced by doses of 3 μg/L and 5 μg/L. GAL, used as a positive reference drug, evoked memory-enhancing effects, as noticed by the behavioral parameters.

**Figure 4:**
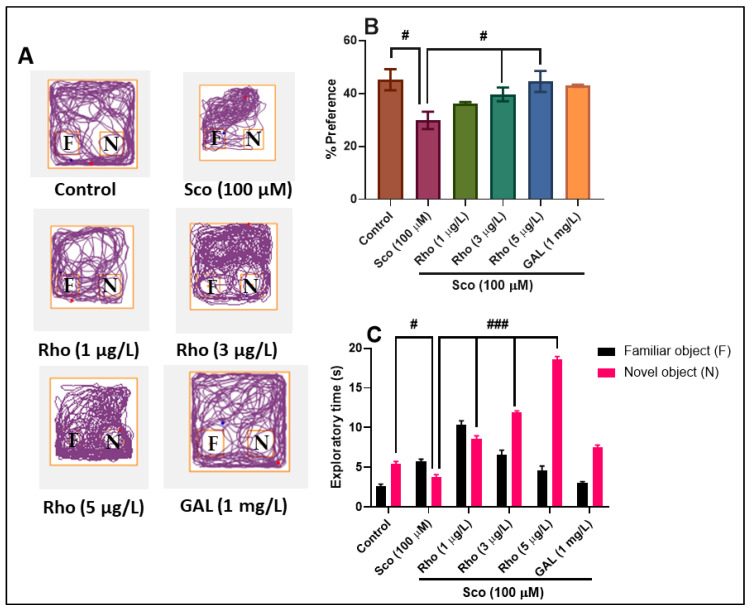
Rhoifolin (Rho: 1, 3, and 5 μg/L) improved memory in the novel object recognition test (NOR). (**A**) Representative locomotion tracking pattern of the control, scopolamine (Sco: 100 µM), rhoifolin (Rho: 1, 3, and 5 µg/L), and galantamine (GAL: 1 mg/L) treated groups. (**B**) Representation of the percentages of preference in different groups. (**C**) Representation of the exploratory time in different groups. Values are means ± S.E.M. (n = 10). For Tukey’s post hoc analyses: (**B**) Control vs. Sco (100 µM): # *p* < 0.01, Sco vs. Rho (3 µg/L): # *p* < 0.01, and Sco vs. Rho (5 µg/L): # *p* < 0.01; (**C**) Control vs. Sco (100 µM): # *p* < 0.01, Sco vs. Rho (1 µg/L): ### *p* < 0.0001, Sco vs. Rho (3 µg/L): ### *p* < 0.0001, and Sco vs. Rho (5 µg/L): ### *p* < 0.0001.

The typical locomotion tracking pattern (Figure 4A) illustrates the differences in the exploration of the familiar object (F) and the novel object (N) within the NOR. It shows that the Sco-treated group exhibited a high preference to explore F, indicating memory deficits. In the NOR test, the one-way ANOVA revealed a significant effect of the treatment on the preference percentages (F (5, 54) = 4.21, *p* < 0.001) (Figure 4B) and on the exploratory time (F (5, 54) = 12.22, *p* < 0.0001) (Figure 4C). Animals treated with Sco showed fewer percentages of preference (*p* < 0.01) (Figure 4B) and exploratory time to explore N (*p* < 0.01) (Figure 4C), as compared with the control group, while the administration of Rho, especially at doses of 3 μg/L and 5 μg/L, improved the preferences and exploratory time for the N, suggesting a memory-enhancing profile. GAL used as a positive reference drug evoked memory-enhancing effects, as noticed by the behavioral parameters, supporting the data delivered by the Y-maze test.

Our results are precisely in line with those obtained by other groups, proving the neuroprotective role of Rho. Hashemi et al. [32] demonstrated that apigenin might have a protective effect on memory deficiency in specific behavioral tasks such as the Y-maze and Morris water maze, caused by kainite through anticonvulsant and anti-apoptosis activity. In another study, Nikbakht et al. [33] reported that apigenin significantly ameliorated spatial working memory impairment induced by Aβ25-35 in the Y-maze test. Chen et al. [34] identified apigenin as a potent suppressor of isoflurane exposure-induced learning and memory dysfunction in rats when tested within the Morris water maze test. Based on these results, Rho could be considered a therapeutic agent with a high potential to improve cognitive deficits.

### 3.2. Effects on the Brain AChE Activity

**Figure 5:**
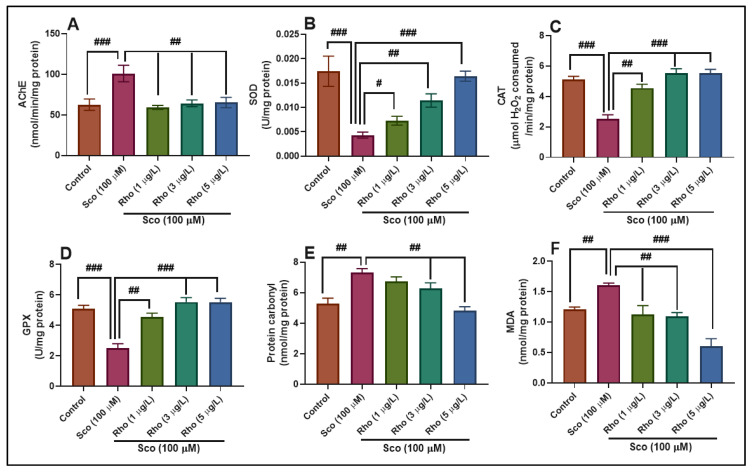
Rhoifolin (Rho: 1, 3, and 5 μg/L) exhibited an anti-AChE effect and improved the antioxidant status in the zebrafish brain. (**A**–**D**) Representation of the enzymes’ specific activity (AChE, SOD, CAT, and GPX) in different groups; (**E**,**F**) Representation of the protein carbonyl and MDA levels in different groups. Values are means ± S.E.M. (n = 10). For Tukey’s post hoc analyses: (**A**) Control vs. Sco (100 µM): ### *p* < 0.0001, Sco vs. Rho (1 µg/L): ## *p* < 0.001, Sco vs. Rho (3 µg/L): ## *p* < 0.001 and Sco vs. Rho (5 µg/L): ## *p* < 0.001; (**B**) Control vs. Sco (100 µM): ### *p* < 0.0001, Sco vs. Rho (1 µg/L): # *p* < 0.01, Sco vs. Rho (3 µg/L): ## *p* < 0.001 and Sco vs. Rho (5 µg/L): ### *p* < 0.0001; **C**. Control vs. Sco (100 µM): ### *p* < 0.0001, Sco vs. Rho (1 µg/L): ## *p* < 0.001, Sco vs. Rho (3 µg/L): ### *p* < 0.0001 and Sco vs. Rho (5 µg/L): ### *p* < 0.0001; (**D**) Control vs. Sco (100 µM): ### *p* < 0.0001, Sco vs. Rho (1 µg/L): ## *p* < 0.001, Sco vs. Rho (3 µg/L): ### *p* < 0.0001 and Sco vs. Rho (5 µg/L): ### *p* < 0.0001; (**E**) Control vs. Sco (100 µM): ## *p* < 0.001, Sco vs. Rho (3 µg/L): ## *p* < 0.001 and Sco vs. Rho (5 µg/L): ## *p* < 0.001; and (**F**) Control vs. Sco (100 µM): ## *p* < 0.001, Sco vs. Rho (1 µg/L): ## *p* < 0.001, Sco vs. Rho (3 µg/L): ## *p* < 0.001 and Sco vs. Rho (5 µg/L): ### *p* < 0.0001.

AChE plays a significant role in the degradation of ACh, a crucial cholinergic neurotransmitter [35]. Sco-administered zebrafish exhibited a significant increase in the AChE activity (*p* < 0.0001) (Figure 5A) when compared to the control group. Rho-treated zebrafish exhibited a significant decrease in the AChE activity (*p* < 0.001) when compared to Sco alone-treated animals, and this could be correlated to the improvement of memory parameters, as evidenced in the behavioral approaches (NTT, Y-maze, and NOR tests). Fan et al. [36] demonstrated the anti-AChE activity of Rho. Additionally, Zhang et al. [37] showed that Rho could inhibit a variety of important enzymes such as aggrecanase, aldose reductase, α-glucosidase, cholinesterase, protein tyrosine phosphatase and AChE, and tyrosinase. Our findings indicate, therefore, that Rho has the potential to improve cognitive dysfunction in Sco-induced amnesic zebrafish, probably by inhibiting AChE activity, as stated in the studies mentioned above.

### 3.3. Effects on Brain Oxidative Status

Sco-induced anxiety and amnesia are closely related to increased oxidative stress in the zebrafish brain. Sco-administered zebrafish clearly showed suppressed antioxidant enzymes SOD (*p* < 0.0001) (Figure 5B), CAT (*p* < 0.0001) (Figure 5C), and GPX (*p* < 0.0001) (Figure 5D) specific activity in the brain along with increased levels of protein carbonyl (*p* < 0.001) (Figure 5E) and lipid peroxidation (MDA) (*p* < 0.001) (Figure 5F) when compared to the control group. Alternatively, Rho treatment inhibited, in a dose-dependent manner, Sco-induced oxidative stress by enhancing the antioxidant enzyme activities and suppressing the protein carbonyl and lipid peroxidation levels when compared to Sco-treated animals.

Accumulating evidence suggests that the oxidative stress produced by the reactive oxygen species (ROS)/reactive nitrogen species (RNS) plays an essential role in the progression of AD in the aging population [35,38]. Besides the behavioral impairment, Sco-induced amnesic zebrafish could mimic the oxidative stress event of AD development, although its exact oxidative damage mechanism is uncertain [39]. Nuclear factor erythroid 2-related factor 2 (Nrf2) is a transcription factor that activates the pathways of endogenous antioxidant defense and the production of the antioxidant enzymes [4,40]. Kanninen et al. [41] showed that attenuation of the Nrf2-ARE pathway coincided with disease progression in APdE9 transgenic mice modeling AD. The authors demonstrated that the Nrf2-pathway was impaired in transgenic mice in a way that paralleled increased brain Aβ burden. Recent studies have shown that the activation of the Nrf2 signaling pathway could enhance cognitive impairment in AD mice models [42,43,44]. Sco incubation at a concentration of 5 mM decreased the protein expression level of Nrf2 by 30% in C6 glioma cells [45]. An animal study on male Wistar rats showed that treatment with Sco at 2 mg/kg/day for six consecutive weeks downregulated the hippocampal transcription factor Nrf2 to 30% [45,46]. Inflammation plays a key role in brain-aging and progressive neurodegenerative disorders, including AD [47]. While microglial activation is a resident innate immune protection in the central nervous system [48], over-activation of microglial cells can trigger inflammatory reactions that produce neurotoxic compounds, including nitric oxide (NO), prostaglandin E_2_ (PGE_2_), and tumor necrosis factor-α (TNF-α) [49]. Besides, it has been shown that Rho regulated oxidative stress and proinflammatory cytokine levels in Freund’s adjuvant-induced rheumatoid arthritis by inhibition of the NF-κB pathway [50]. As expected in this study, the administration of Sco significantly increased levels of protein carbonyl and lipid peroxidation (MDA), suggesting the induction of oxidative stress in the zebrafish brain. Rho successfully reversed this change in the zebrafish brain, which is consistent with the findings of previous studies [6,51,52]. Cellular organisms typically retain an effective integrated antioxidant protection system, which consists of enzymatic and non-enzymatic factors, to protect tissue from the impact of oxidative stress [53]. In this study, the protective functions of the endogenous antioxidant enzymes SOD, CAT, and GPX were remarkably suppressed in the Sco-treated zebrafish. The suppression of the antioxidant enzyme activities may lead to high levels of free radicals’ accumulation in the cell, such as ROS. Indeed, Sco also induces ROS accumulation [54]. Alternatively, Rho treatment effectively restored the antioxidant defense mechanism by increasing the antioxidant levels of activity in the brain. Additionally, the antioxidant action of Rho was probably mediated by the activation of the Nrf2 signaling pathway and could ameliorate the observed cognitive deficits in the Sco-induced amnesic zebrafish model.

## 4. Conclusions

By using spectroscopic methods, the structure of Rho was elucidated. Rho could effectively improve memory impairments in a Sco-induced zebrafish model by enhancing the function of the cholinergic system and via upstream antioxidant enzymes in the amnesic zebrafish model. The findings that were obtained illustrate the possible health benefits from Rho being investigated and indicate its possible use in formulating new medicines for the amelioration of dementia.
